# Changing epidemiology of leptospirosis in China from 1955 to 2022

**DOI:** 10.1186/s40249-025-01284-x

**Published:** 2025-03-03

**Authors:** Zengliang Wang, Ke Li, Yuanhua Liu, Michael P. Ward, Yue Chen, Shuting Li, Jidan Zhang, Yu Zhao, Na Wang, Haiyan Qiu, Yueran Lian, Cuicai Zhang, Zhijie Zhang, Biao Kan

**Affiliations:** 1 Department of Epidemiology, School of Public Health, Cheeloo College of Medicine, Shandong University, Jinan, Shandong China; 2 Shanghai Institute of Infectious Disease and Biosecurity, Fudan University, Shanghai, China; 3 Department of Epidemiology and Health Statistics, School of Public Health, Fudan University, Shanghai, China; 4 Key Laboratory of Public Health Safety, Ministry of Education, Shanghai, China; 5 Sydney School of Veterinary Science, The University of Sydney, Camden, Sydney, NSW Australia; 6 School of Epidemiology and Public Health, Faculty of Medicine, University of Ottawa, Ottawa, ON Canada; 7 National Key Laboratory of Intelligent Tracking and Forecasting for Infectious Diseases, National Institute for Communicable Disease Control and Prevention, Chinese Center for Disease Control and Prevention, Beijing, China

**Keywords:** Leptospirosis, Epidemiology, Climate, Socioeconomics, China

## Abstract

**Background:**

Leptospirosis, a zoonotic disease caused by pathogenic species of the genus *Leptospira*, is an important public health concern globally. Leptospirosis has been notifiable under statute in China since 1955, and its epidemiological characteristics have evolved during near 70 years. This study aimed to describe the spatial and temporal patterns and demographic characteristics of leptospirosis from 1955 to 2022 in China, and explore the possible factors that influence leptospirosis transmission risk.

**Methods:**

Wavelet time series analysis, global Moran’s *I* coefficients, space–time scanning statistics, and so on were used to analyze temporal, seasonal, geographic, and demographic trends in leptospirosis using reported national surveillance data from Chinese mainland from 1955 to 2022. Additionally, a Bayesian spatiotemporal model was used in a preliminary analysis to explore potential factors associated with leptospirosis occurrence.

**Results:**

Between 1955 and 2022, China reported 25,236,601 leptospirosis cases, with 91% occurring from July to October. The annual incidence rate peaked at 38.28/100,000 during outbreaks in the 1960s–1980s but stabilized at a low level (0.07/100,000) between 2005 and 2022, with over 99% of cases in southern China. Clustering increased over time, being greatest during the period 2015–2022 (Moran’s *I* = 0.41, *P* < 0.01). Space-time cluster analysis indicated that the most likely clusters were in northern provincial-level administrative divisions (PLADs) from 1955 to 1984, in southern PLADs from 1985 to 2022. The main identified risk factors of leptospirosis occurrence were annual average precipitation (3.68, 95% *CI:* 2.50 to 5.12), GDP per capita (-3.70, 95% *CI:* − 5.97 to − 1.41), and the total power of agricultural machinery (− 2.51, 95% *CI:* − 3.85 to − 1.17).

**Conclusions:**

Over past 70 years, leptospirosis in China has occurred as significant outbreaks but has ultimately declined to stable, low levels of occurrence. However, a clear north–south disparity persists, with tropical and subtropical regions in southern China remaining high-risk areas. The nearly 70-year dataset underscores the complex interplay of climate and socioeconomic factors influencing the disease’s occurrence. Targeted prevention and control measures are critical to prevent outbreaks, especially in regions prone to extreme climatic events like heavy rainfall and floods, which may signal the resurgence of leptospirosis.

**Supplementary Information:**

The online version contains supplementary material available at 10.1186/s40249-025-01284-x.

## Background

Leptospirosis is a zoonotic disease of global public health significance, primarily affecting tropical and subtropical regions. It is caused by pathogenic species of the genus *Leptospira *[1–4]. Over 200 species of animals − including fish, amphibians, reptiles, birds, and mammals − serve as hosts for pathogenic leptospires in nature [2, 5]. Among these, rodents and certain domestic animals (such as pigs, cattle, and dogs) are considered the primary reservoirs, posing the greatest risk of transmission to humans [6]. Infected animals can excrete leptospires in their urine for extended periods, sometimes throughout their lifetime, leading to contamination of natural water sources via soil and creating persistent infection risks. Human infection typically occurs through direct or indirect contact with the urine or body fluids from carrier animals or through the ingestion of contaminated food or water [7]. Globally, at least 24 serogroups and more than 300 serotypes of *Leptospira* have been identified [3, 5]. In China, more than 70 serotypes from 18 serogroups have been documented, with *L.* *interrogans* serogroup Icterohaemorrhagiae serovar Lai being the most prevalent [8]. Other common pathogens include Grippotyphosa, Autumnalis, Australis, Pomona and Hebdomaidis [9].

Leptospirosis is widely distributed and is endemic in many parts of the world. According to the World Health Organization (WHO), approximately one million cases of leptospirosis occur annually worldwide, with about 60,000 deaths [10]. In recent years, the risk of leptospirosis outbreaks has markedly increased due to global warming and extreme weather events, particularly flooding events [11, 12]. This has led to a rise in leptospirosis incidence in regions such as Southeast Asia and South America, but increased incidence has also been reported in European and North American countries [10, 13, 14]. Leptospirosis is a re-emerging disease of significant global public health concern, contributing to substantial morbidity and mortality in both humans and animals [15]. Factors such as lack of treatment, rapid and unplanned urbanization, and inadequate sanitation have led to leptospirosis becoming a leading cause of acute febrile illness in many developing countries [11, 15].

Historically, leptospirosis was a serious public health problem in China, particularly in the southwest and southeastern regions. The first case of leptospirosis in China was reported in 1937 in Guangzhou, Guangdong Province,. In the 1950s, *Leptospira* was subsequently isolated from patients in several regions, including Guangdong, Zhejiang, and Yunnan provinces. Leptospirosis was officially classified as a notifiable infectious disease in China in 1955. In the ensuing decades, the country experienced numerous large-scale leptospirosis outbreaks, which posed a serious threat to public health and the safety of the Chinese population [16]. Based on epidemiological characteristics, leptospirosis in China manifests in three primary epidemic forms: (1) In the Yangtze River Basin and its southern regions, cases are primarily associated with paddy field labour or the reclamation of wasteland and swamps, with rats serving as the main source of infection. (2) In the Yellow River Basin and northern China, infections are frequently linked to flood exposure, with pigs and dogs identified as the primary reservoirs. (3) In low-lying plains, heavy rainfall often leads to waterlogging, which facilitates the transmission of leptospirosis [8].

Leptospirosis transmission is promoted by climatic variables such as heavy rain, floods, humidity and high temperatures [3, 17, 18]. The influence of meteorological factors on the incidence of leptospirosis has been extensively investigated in different geographical and climatic zones [17–21]. Previous research demonstrated strong associations between leptospirosis incidence and climatic factors [18]. For instance, in Puerto Rico, leptospirosis outbreaks were influenced by Hurricane Fiona, which caused heavy rainfall and severe flooding [22]. Studies have also found that human leptospirosis risk is associated with temperature[23] and socioeconomic variables [18, 24].

To date, few studies have investigated the changing epidemiology of leptospirosis in China [16, 25]. Existing studies have only provided simple descriptions of the epidemiological characteristics or have been limited to local areas. There is a lack of high-quality, nationwide epidemiological studies. In addition, the reasons behind the changing epidemiology of leptospirosis in China since 1955 remain unclear. This study aimed to (i) describe the spatial and temporal patterns and demographic characteristics of leptospirosis from 1955 to 2022 in China, and (ii) explore the possible factors that influence leptospirosis transmission risk.

## Methods

### Case data

Since 1955, leptospirosis has been legally classified as a Class B Notifiable Disease in China, with aggregated data for each provincial-level administrative divisions (PLADs) were required to be reported. In 2004, the real-time online National Notifiable Infectious Disease Reporting Information System (NIDRIS) was implemented, requiring all notifiable diseases to be reported within 24 h [26–28]. Due to changes in reporting requirements over time, this study utilized two datasets. Aggregated leptospirosis data from 1955 to 2004 were obtained from the infectious disease history database of the Public Health Science Data Center at the Chinese Center for Disease Control and Prevention (China CDC). The individual case data from 2005 to 2022 were sourced from NIDRIS. The aggregated data include the numbers of cases and deaths, incidence rates and case-fatality rates at the national and provincial levels (Additional file: Table S1). Individual data include variables such as gender, age, occupation, zone code of current address, and case classification (suspected, clinically diagnosed or confirmed). All data used in this study were anonymized to ensure that individual patients could not be identified.

The study area encompasses 31 PLADs in the Chinese mainland, which includes 22 provinces, four municipalities, and five autonomous regions, not including Hong Kong, Macao, and Taiwan. Administrative adjustments during the study period include Hainan becoming a province in 1988 and Chongqing becoming a directly-administered municipality in 1997. Historical data for Hainan and Chongqing before these changes are aggregated within Guangdong and Sichuan, respectively.

### Case definition

Leptospirosis cases were classified as suspected, clinically diagnosed and laboratory-confirmed based on the diagnostic criteria established in 1996 and updated in 2008 (GB 15995–1995; WS 290–2008), incorporating the patients’ epidemiological history, clinical presentations, and laboratory findings[25, 29–31].

Suspected cases are defined as individuals with a history of exposure to contaminated water, or to the urine or blood of animals carrying *Leptospira* within 1–30 days before symptom onset, accompanied by at least one of the following symptoms: (1) fever, (2) myalgia, or (3) fatigue.

Clinically diagnosed cases include suspected cases that present with at least one of the following additional symptoms: (1) conjunctival congestion, (2) gastrocnemius muscle tenderness, and (3) lymphadenopathy.

Laboratory-confirmed cases are suspected cases with evidence of one or more of the following laboratory findings: (1) isolation of *Leptospira* from blood, cerebrospinal fluid, or urine, (2) detection of *Leptospira* nucleic acid in blood, cerebrospinal fluid, or urine, (3) a four-fold or greater increase in serum antibody titers between acute and convalescent samples, or a single serum antibody titer of ≥ 1:400.

### Geographic, climate and socioeconomic data

To identify potential factors influencing transmission risk for leptospirosis in China during the past 68 years, we collected province-level geographic, climate and socioeconomic data. Climate data − including annual average temperature (℃), annual average precipitation (mm), relative humidity (%) and daily sunshine hours (h) − were obtained from the National Climatic Data Center (NCDC, ftp://ftp.ncdc.noaa.gov/pub/data/noaa/isd-lite/). Elevation data were downloaded from the GEBCO Compilation Group (2024) GEBCO 2024 Grid (https://www.gebco.net/data_and_products/gridded_bathymetry_data/). Socioeconomic data − including annual gross domestic product (GDP) per capita (Chinese Yuan, CNY), total power of agricultural machinery (10,000 kw), and population − were retrieved from the China Statistical Yearbook (https://www.stats.gov.cn/sj/ndsj/). To eliminate the effect of the time value of money, the GDP per capita for each PLAD was adjusted using a discount rate of 3% per year, as recommended by the WHO Guidelines on Health Economics[32].

## Data analysis

Our study included all clinically diagnosed and laboratory-confirmed cases of leptospirosis from 1955 to 2022. Each PLAD was categorized as either a temperate northern province (16 PLADs) or a subtropical southern province (15 PLADs) based on a previous study[33]. This classification allowed for the comparison of spatial and temporal patterns, as well as demographic characteristics of leptospirosis cases, between northern and southern China.

To visualize the characteristics of long-term changes and seasonal distribution at the provincial level, we created heat maps that displayed yearly and monthly incidence rates. To analyze the periodicity of leptospirosis in the endemic PLADs (cases ≥ 100), we performed wavelet time series analysis on monthly cases from 1980 to 2022, in which all series were log-transformed and scaled to have a mean of zero and a variance of one.

Global Moran’s *I* coefficients were calculated to assess the overall spatial autocorrelation of the average incidence rate across 10-year periods from 1955 to 2022. Retrospective space-time scanning statistics, based on a discrete Poisson distribution, were applied to analyze the temporal and spatial dynamics of case distributions across 31 PLADs in 10-year periods from 1955 to 2022. We set the maximum scanning window base to include up to 10% of the total population to avoid the potential of detecting extremely large clusters that are difficult to interpret, and the upper temporal bound to 50% of the entire study period.

To identify potential influencing factors for leptospirosis at the provincial level, we developed a spatiotemporal model within a Bayesian framework using Integrated Nested Laplace Approximation (INLA). The independent variables included GDP per capita, the total power of agricultural machinery, elevation, annual average temperature, annual average precipitation, relative humidity and daily sunshine hours, to represent socioecomonic and climate factors. All data were normalized to eliminate the influence of dimension. Spearman’s correlation coefficients were used to examine the bivariate association between independent variables. Strongly correlated variables (*r* ≥|0.7|)[19] were not included in the model to avoid multicollinearity issues. The model formulas were as follows:

$$Y_{ij} \sim Poisson\left( {\lambda_{it} } \right)$$

$$\lambda it = Eit\theta it$$

$$log(\theta it) = b0 + ui + vi + \sum k \beta kXkit + \gamma t + \phi t + \delta it$$

where *i* = 1, 2, …, 31, *t* = 1, 2, …, 68, *k* = 1, 2, …, 4. *Y*_*it*_ is the observed count of leptospirosis cases in the *i*th PLAD and *t*th year, following the Poisson distribution with a mean value of *λ*_*it*_; *E*_*it*_ is the expected number of leptospirosis cases in the PLAD *i* and year *t*;*θ*_*it*_ is the relative risk; *b*_*0*_ is the average *log* relative risk; *u*_*i*_ is the spatial structured effect of the PLAD *i*; *v*_*i*_ is the spatial unstructured effect of the PLAD *i*; *X*_*kit*_ is the value of the *k*th risk factor in year *t* of PLAD *i*; *β*_*k*_ represents the effect of the *k*th influencing factor;*γ*_*t*_ is the structured effect of the year *t*. *ϕ*_*t*_ is the unstructured effect of the year* t*; *γ*_*t*_ and *ϕ*_*t*_ can be regarded as hidden variables of the year *t*, which are related to and irrelevant to the position of year *t*; and *δ*_*it*_ is the spatial–temporal interaction effect in the PLAD *i* and year *t*.

The statistical analyses were conducted using R 4.4.1 (R Foundation for Statistical Computing, Vienna, Austria). Graphs and heatmaps were created using *ggplot2* and *sf* packages. Wavelet analysis was performed using the *WaveletComp* package. The spatiotemporal model developed within INLA was implemented through the *R-INLA* package. Additionally, the SaTScan v10.1 (Martin Kulldorff together with Information Management Services Inc, Maryland, USA) was used to analyze the space-time cluster.

## Results

### Temporal trend and seasonality of leptospirosis in China

**Fig. 1:**
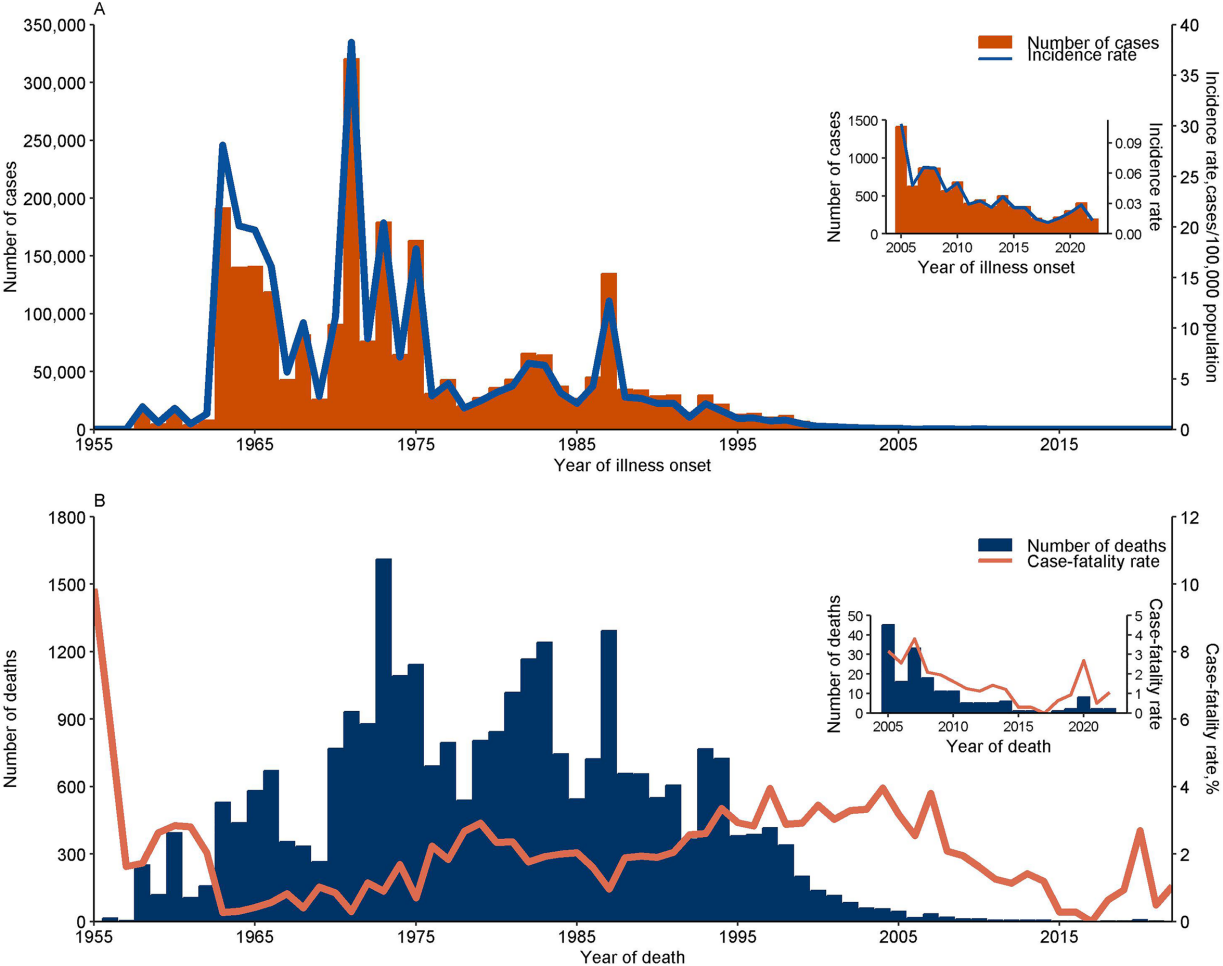
Reported leptospirosis cases and deaths, China, 1955–2022. A: Number of leptospirosis cases and incidence rate (no. cases/100,000 population) by year. B: Number of leptospirosis deaths and case-fatality rate (%) by year

Between 1955 and 2022, a total of 2,523,601 cases of leptospirosis were reported in China, including 27,734 (1.1%) deaths. The incidence rate was relatively low in the 1950s (1955–1959), with an average of 0.61/100,000 per annum. In the 1960s and 1970s, the average annual incidence rate of leptospirosis sharply increased to 11.23/100,000 (range: 0.58–38.28/100,000). During the 1980s, the average annual incidence rate was 5.03/100,000, with a notable outbreak in 1987, when the incidence rate reached 12.69/100,000. The epidemic gradually declined after the 1990s, dropping from 2.59/100,000 to 0.32/100,000 by 2000. Since then, the epidemic stabilized at a low level, with an average incidence rate of 0.07/100,000 (Fig. 1A). The case-fatality rate was as high as 9.87% in 1955, then fluctuated over time (Fig. 1B).

**Fig. 2:**
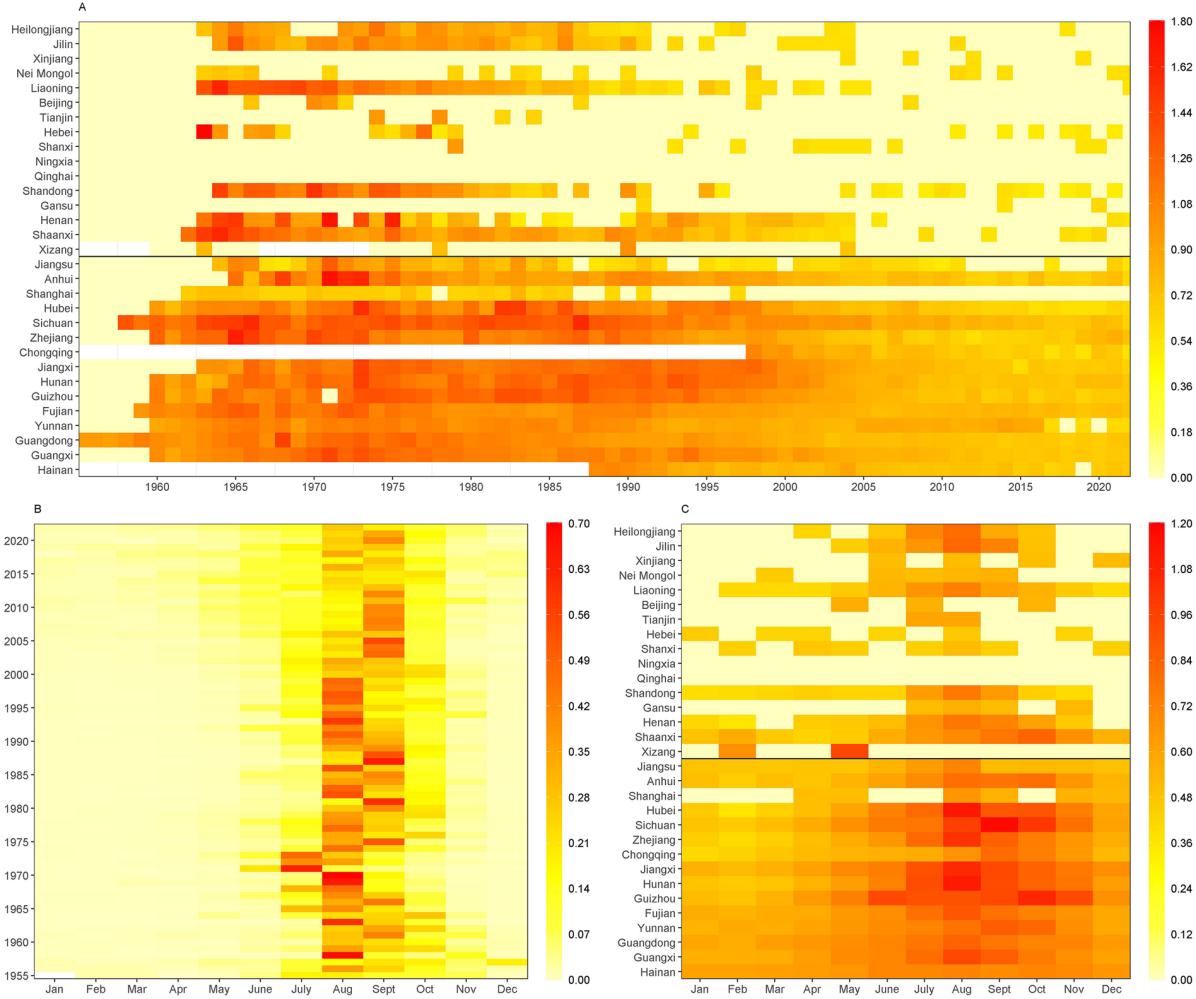
Heat map of leptospirosis in China. A: Time series of incidence rate per 100,000 population during 1955–2022, standardized by the ten roots. B: Seasonal distribution of cases by year, plotted as the proportion of cases in each month of the year from 1955 through 2022. C: Seasonal distribution of cases by province, plotted as the mean value of incidence rate per 100,000 population in each month of the year from 1980 through 2022, standardized by the ten roots

Leptospirosis cases occurred throughout the year, with peak epidemics during the summer and autumn, particularly from July to October, accounting for 91% of total cases (Fig. 2B). Wavelet time series analysis revealed that both local (Additional file: Fig S1) and global (Additional file: Fig S2) wavelet powers were highest at 1 year in Heilongjiang, Jilin, Liaoning, Shandong, Henan, Shaanxi, Anhui, Hubei, Hunan, Guizhou, Fujian, Yunnan, Guangdong, and Guangxi. This indicates that leptospirosis cases in these PLADs exhibit a single annual peak, with stable seasonal fluctuations maintained over 12-month periods, as evidenced by the continuous red region at the 1 year in both maps. In contrast, Jiangsu and Hainan did not display a significant 1-year periodicity.

However, the months of peak onset varied across PLADs. From 1980 to 2022, most PLADs − including Guangdong, Fujian, Jiangxi, Hubei, Hunan, Zhejiang, Heilongjiang, Jilin, and Shanxi − experienced peak cases in August. Whereas the peak occurred in September in Sichuan and Chongqing. Hainan, a tropical province, had the highest incidence from May to October, accounting for 76.02% of annual cases. Notably, both Shaanxi and Guizhou experienced peaks in October (Fig. 2C).

### Geographic distribution of leptospirosis in China

**Fig. 3:**
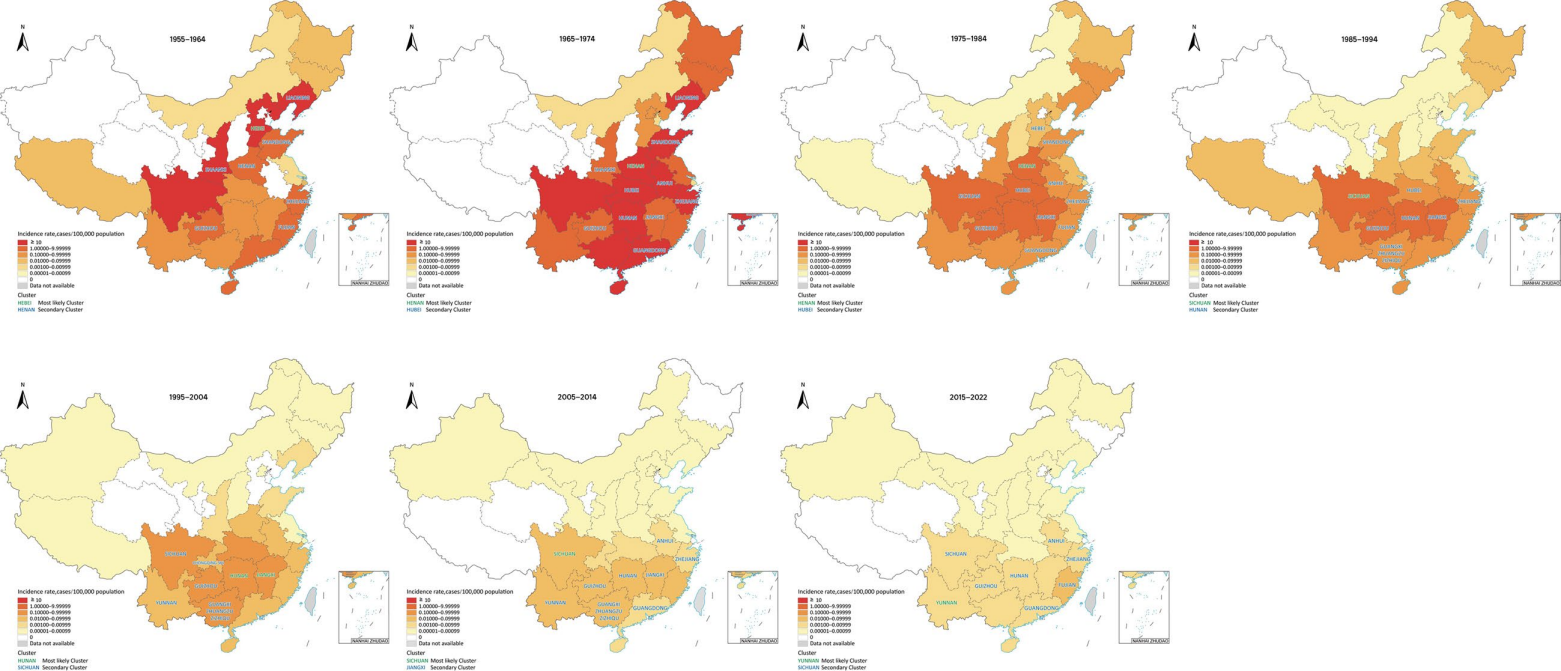
Geographic distribution and Space–time cluster analyses of leptospirosis by 10-year periods, China, 1955–2022. Map approval number: GS(2005)0700

From 1955 to 2022, leptospirosis cases were reported in 29 PLADs across the Chinese mainland, except for Ningxia and Qinghai (Fig. 2A). The epidemic became widespread in the late 1950s, particularly in southern PLADs along the Yangtze River basin. By the 1960s, cases were reported from all PLADs except for Qinghai, Gansu, Ningxia, Xinjiang, and Tianjin. Most PLADs experienced several outbreaks between the 1960s and 1980s, followed by a gradual decline in incidence rates after the 1990s. In the twenty-first century, the intensity and geographic distribution of the epidemic further diminished and stabilized. From 1955 to 2004, the PLADs with the highest median incidence rates were Sichuan (0.80/100,000), Jiangxi (4.31/100,000), Hunan (4.31/100,000), Guizhou (4.04/100,000), and Zhejiang (2.18/100,000), with southern PLADs accounting for 60% of the total cases nationwide. In the period 2005–2022, the PLADs with the highest median incidence rates were Yunnan (0.21/100,000), Fujian (0.09/100,000), Sichuan (0.08/100,000), Hunan (0.05/100,000), and Guangdong (0.04/100,000), with the proportion of cases in southern PLADs rising to 99% (Fig. 2A, Fig. 3).

The Moran’s *I* value revealed a significant (*P* < 0.05) positive spatial autocorrelation, except for 1955–1964, demonstrating a non-random distribution over time. Additionally, there was an increasing trend in Moran’s *I* value over time, peaking at 0.41 (*P* < 0.01) in 2015–2022, suggesting a growing degree of spatial clustering in incidence rates (Table 1).

**Table 1:** Spatial autocorrelation (Global Moran’s *I*) of human leptospirosis in China from 1955–2022

Year	Moran’s *I*	*P*	Z
1955–1964	− 0.02	0.44	0.15
1965–1974	0.21	0.01	2.19
1975–1984	0.26	< 0.01	2.63
1985–1994	0.29	< 0.01	3.11
1995–2004	0.33	< 0.01	3.46
2005–2014	0.33	< 0.01	3.38
2015–2022	0.41	< 0.01	3.77

Space-time cluster analyses identified the most likely cluster in the northern region (e.g., Hebei in 1963 and Henan in 1971/1975) during the 1950s–1980s and in the southern region (e.g., Sichuan in 1987 and 2005–2009, Hunan and Jiangxi in 1995–1999, Yunnan in 2015–2016) from the 1980s to the 2020s (Fig. 3, Additional file: Table S2).

### Demographic features of human leptospirosis in China

From 2005 to 2022, 8967 confirmed human leptospirosis cases were reported, with 99% occurring in southern China. The disease affected all age groups, with a median age of 45 (31.00, 57.00) years. Males consistently made up approximately 70% of cases. Farmers represented the majority of cases (> 70%), followed by individuals in other occupations and students (Table 2).

**Table 2:** Demographic characteristics of patients with human leptospirosis, categorised by northern and southern China, 2005–2022

Characteristic	Northern	Southern	Total*
	(*n* = 76)	(*n* = 8868)	(*n* = 8967)
**Gender**			
Male	46 (60.53)	6206 (69.98)	6268 (69.90)
Female	30 (39.47)	2662 (30.02)	2699 (30.10)
**Age, years**			
Median(Q1, Q3)	48 (32.00, 58.25)	45 (31.00, 57.00)	45 (31.00, 57.00)
**Age group**			
0–4	2 (2.63)	18 (0.20)	20 (0.22)
5–14	1 (1.32)	550 (6.20)	551 (6.14)
15–24	6 (7.89)	950 (10.71)	960 (10.71)
25–34	13 (17.11)	1182 (13.33)	1196 (13.34)
35–44	11 (14.47)	1718 (19.37)	1731 (19.30)
45–54	15 (19.74)	1784 (20.12)	1807 (20.15)
55–64	17 (22.37)	1618 (18.25)	1640 (18.29)
≥ 65	11 (14.47)	1048 (11.82)	1062 (11.84)
**Occupation**			
Farmer	44 (57.89)	6582 (74.22)	6644 (74.09)
Student	1 (1.32)	757 (8.54)	760 (8.48)
Worker	3 (3.95)	266 (3.00)	269 (3.00)
Others	25 (32.89)	1121 (12.64)	1149 (12.81)
Unknown	3 (3.95)	142 (1.60)	145 (1.62)

*The Total includes 23 cases without address information

### Factors influencing transmission risk for leptospirosis epidemic in China

Spearman correlation analysis revealed no strong correlation between GDP per capita, the total power of agricultural machinery, elevation, and other factors (*r* < 0.40) (Additional file: Table S3). Based on previous literature[18, 19, 24, 34, 35], meteorological covariates with greater biological relevance were retained in cases in which variables were strongly correlated (*r* > 0.7). The final Bayesian spatiotemporal model included four factors: GDP per capita, the total power of agricultural machinery, annual average precipitation and elevation. GDP per capita(-3.70, 95% *CI:* −5.97 to −1.41), total power of agricultural machinery(-2.51, 95% *CI:* −3.85 to −1.17) and elevation(-7.51, 95% *CI:* −12.67 to −2.70) showed significant negative associations with the incidence of leptospirosis. In contrast, increases in annual average precipitation (3.68, 95% *CI:* 2.50 to 5.12) were significantly positively associated with the incidence of leptospirosis (Table 3).

**Table 3:** Posterior estimates [mean, standard deviation (SD), and quantiles] for the fixed effects of potential factors influencing transmission risk of leptospirosis

Variables	Mean	SD	2.5%	50%	97.5%
GDP per capita	− 3.70	1.16	− 5.97	− 3.70	− 1.41
Total power of agricultural machinery	− 2.51	0.68	− 3.85	− 2.51	− 1.17
Elevation	− 7.51	2.53	− 12.67	− 7.45	− 2.70
Annual average precipitation	3.68	0.73	2.25	3.68	5.12

## Discussion

This study examined the epidemiological trends and potential factors influencing transmission risk for leptospirosis in China for nearly 70 years. We observed a significant overall decline in the incidence of leptospirosis, alongside marked spatial and temporal variations across PLADs between 1955 and 2022. High-risk areas persisted in the tropical and subtropical impoverished regions of southern China. Climate and socioeconomic factors mostly influence this disease occurrence, with certain factors, such as floods, acting as key indicators for monitoring the potential resurgence of leptospirosis in at-risk areas. These findings are expected to provide valuable insights for future research and efforts to control leptospirosis in China and other leptospirosis-endemic regions.

Since its classification as a legally reportable infectious disease in 1955, China has experienced numerous large-scale epidemics, most driven by floods[8]. Notable examples include outbreaks in southern Hebei (1963, incidence rate: 296/100,000), Sichuan (1966, incidence rate: 99/100,000), Anhui (1971, incidence rate: 256/100,000), Henan (1971, incidence rate: 246/100,000; 1975, incidence rate: 136/100,000), primarily due to torrential rains and flooding[8]. These conditions facilitated the occurrence of leptospire-contaminated water, exposing individuals to flood-related activities and crop harvesting. Our spatiotemporal analysis confirmed that annual average precipitation is positively associated, and elevation is negatively associated, with leptospirosis risk. These findings underscore that flood-prone, low-lying areas are at heightened risk of transmission, aligning with prior studies that highlight the pivotal role of extreme climatic events in triggering leptospirosis outbreaks[17, 24, 36–38]. The potential for leptospirosis re-emergence increases during heavy rainfall and flood events. For instance, Lezhi County, China (2010) [39], Kerala, India (2017–2019) [34], and Fiji (2012) [21] experienced outbreaks of leptospirosis due to flooding. Therefore, targeted interventions, such as improving the drainage systems for better access to safe water and better sanitation and hygiene, are crucial in areas at high risk of flooding [24, 40].

Preventive measures implemented in the 1980s and 1990s, including widespread vaccination and sanitation improvements in livestock husbandry, contributed to stabilizing and reducing the epidemic [8, 30, 41, 42]. During this period, China's socioeconomic status experienced rapid growth following the 1978 reform and opening-up, leading to improved public health infrastructure [43, 44]. Our spatiotemporal model indicated that socioeconomic variables, such as GDP per capita and gross power of agricultural machinery, were negatively associated with leptospirosis incidence. These results are consistent with previous research linking poverty and inadequate sanitation to higher risk, while regions with higher GDP typically benefit from better healthcare and reduced exposure risks due to mechanized farming [24, 45, 46]. Additionally, with the development of the economy, the degree of agricultural mechanization in China’s rural areas has increased. The mechanization process in rural areas where leptospirosis is prevalent has substantially altered farming practices, thereby greatly reducing the risk of farmers being exposed to *leptospira*-contaminated water or soil [36, 47]. A notable peak occurred in 1987, driven primarily by a leptospirosis outbreak in Sichuan, where farmers were exposed while harvesting rice in late August and September [8]. A total of 102,872 cases were reported during this outbreak, with an incidence rate of 99/100,000, accounting for 76% of the total cases in China that year.

In the last two decades, the nationwide incidence of leptospirosis has declined to a lower, stable, and controllable level [48]. Case numbers have fluctuated between 150 and 1800 annually, with sporadic cases being the predominant form of occurrence. Global autocorrelation and spatio-temporal cluster analyses showed an increasing trend in the clustering of leptospirosis over time, with high-risk areas in the southern regions. This pattern suggests a north–south disparity, despite the overall decline in incidence in recent decades.

Historically, leptospirosis was widespread in China, particularly in low-lying hills, basins, and plains, with arid and high-altitude areas largely unaffected. However, over the last two decades, the disease has become confined to southern tropical and subtropical regions, with more than 99% of cases reported from over 100 counties in PLADs like Guangdong, Guangxi, Zhejiang, Sichun, Yunnan, and so on. This change in spatial distribution likely reflects a complex interplay of local epidemiological factors, host animals, epidemic strains, agricultural production activities, socioeconomic factors, and climatic conditions [36, 49]. In the northern regions, infections are often associated with floods caused by heavy rain and the overflow of contaminated livestock waste, leading to human contact with contaminated water [8, 16]. With strengthened prevention and control measures, such as shifting from free-range to confined livestock, and improved sanitation, the chances of livestock in northern regions contracting leptospirosis have significantly decreased, reducing the bacterial carriage rates or eliminating it altogether [8]. In some areas, livestock are no longer the primary source of infection. For example, pigs were the main source of infection in Henan and Shandong provinces before the 1990s, but rodents have since become the primary source [8]. However, in the Yangtze River basin and areas to the south, cases are primarily associated with paddy field labour or land reclamation and marshland work, with rodents being the main source of infection [8, 16]. Farmers are exposed to leptospire-contaminated environments during rice transplanting, field management, and rice harvesting, which corresponds to the demographic characteristics showing that farmers (74%) and males (nearly 70%) account for the majority of cases. Additionally, most southern regions in China have climatic conditions favourable for leptospire survival, with a high diversity of host animals and a high bacterial carriage rate in endemic areas, leading to the long-term persistence of natural foci [50]. One study systematically reviewed the *Leptospira* positivity rates in rodents in China, showing that southern PLADs (Guangxi: 35.29%; Guangdong: 15.17%; Anhui: 15.46%; Zhejiang: 13.70%; Hubei: 10.81%; Yunnan: 9.86%; Jiangxi: 7.23%; Hunan: 7.36%; Sichuan: 6.92%) had generally higher rates than northern PLADs (Henan: 3.61%; Heilongjiang: 1.11%) [51]. Therefore, the incidence of leptospirosis is generally higher in southern than in northern regions.

Despite the declining trend, high-risk populations, particularly those engaged in fieldwork, livestock processing, and outdoor activities, often lack awareness of protective measures, increasing their susceptibility [15, 16]. Additionally, recent global trends, such as an increase in leptospirosis among travellers and water sports participants, highlight the need for ongoing vigilance, especially during heavy rainfall or flooding events [15]. Leptospirosis remains a public health threat and should not be overlooked as an infectious disease.

Our study has several limitations. First, the data were obtained from passive public health surveillance, which may be subject to changes in reporting protocols or underreporting. Second, no data on individual cases were available before 2005, limiting our analysis of demographic characteristics to the period from 2005 to 2022. Third, data on *Leptospira* strains, serotyping, and the clinical manifestations of confirmed cases were not available, preventing us from exploring the relationship between pathogen distribution, reservoir species and disease severity. Nevertheless, the incidence data in this study represent some of the most comprehensive and high-quality national datasets on human leptospirosis in China.

## Conclusions

Over the past 70 years, leptospirosis in China has occurred as significant outbreaks but has ultimately declined to stable, low levels. However, a clear north–south disparity persists, with tropical and subtropical regions in southern China remaining high-risk areas. The nearly 70-year dataset underscores the complex interplay of climate and socioeconomic factors influencing the disease’s occurrence. Targeted prevention and control measures are critical to prevent outbreaks, especially in regions prone to extreme climatic events like heavy rainfall and floods, which may signal the resurgence of leptospirosis.

## Supplementary Information

Supplementary Material 1.

Supplementary Material 2.

Supplementary Material 3.

## Data Availability

Relevant data are available from the corresponding author on request.

## Abbrevations

GDP: Gross domestic product
PLADs: Provincial-level administrative divisions
NIDRIS: National Notifiable Infectious Disease Reporting Information System
China CDC: Chinese Center for Disease Control and Prevention
NCDC: National Climatic Data Center
INLA: Integrated Nested Laplace Approximation
SD: Standard deviation
CI: Credible interval
