# Correction: Changing epidemiology of leptospirosis in China from 1955 to 2022

**DOI:** 10.1186/s40249-025-01313-9

**Published:** 2025-07-10

**Authors:** Zengliang Wang, Ke Li, Yuanhua Liu, Michael P. Ward, Yue Chen, Shuting Li, Jidan Zhang, Yu Zhao, Na Wang, Haiyan Qiu, Yueran Lian, Cuicai Zhang, Zhijie Zhang, Biao Kan

**Affiliations:** 1 Department of Epidemiology, School of Public Health, Cheeloo College of Medicine, Shandong University, Jinan, Shandong China; 2 Shanghai Institute of Infectious Disease and Biosecurity, Fudan University, Shanghai, China; 3 Department of Epidemiology and Health Statistics, School of Public Health, Fudan University, Shanghai, China; 4 Key Laboratory of Public Health Safety, Ministry of Education, Shanghai, China; 5 Sydney School of Veterinary Science, The University of Sydney, Camden, Sydney, NSW Australia; 6 School of Epidemiology and Public Health, Faculty of Medicine, University of Ottawa, Ottawa, ON Canada; 7 National Key Laboratory of Intelligent Tracking and Forecasting for Infectious Diseases, National Institute for Communicable Disease Control and Prevention, Chinese Center for Disease Control and Prevention, Beijing, China

**Correction: Infectious Diseases of Poverty (2025) 14:17** 10.1186/s40249-025-01284-x

Following publication of the original article [1], the authors identified errors in Fig. 1 and Table 2.

In Fig. 1, thousand-separator commas for values < 10,000 were removed to align with journal style guidelines for small integers; axis title was revised from “Yearly number of cases” to “Number of cases” to eliminate redundancy in temporal context.

Fig. 1 was updated from:

**Fig. 1:**
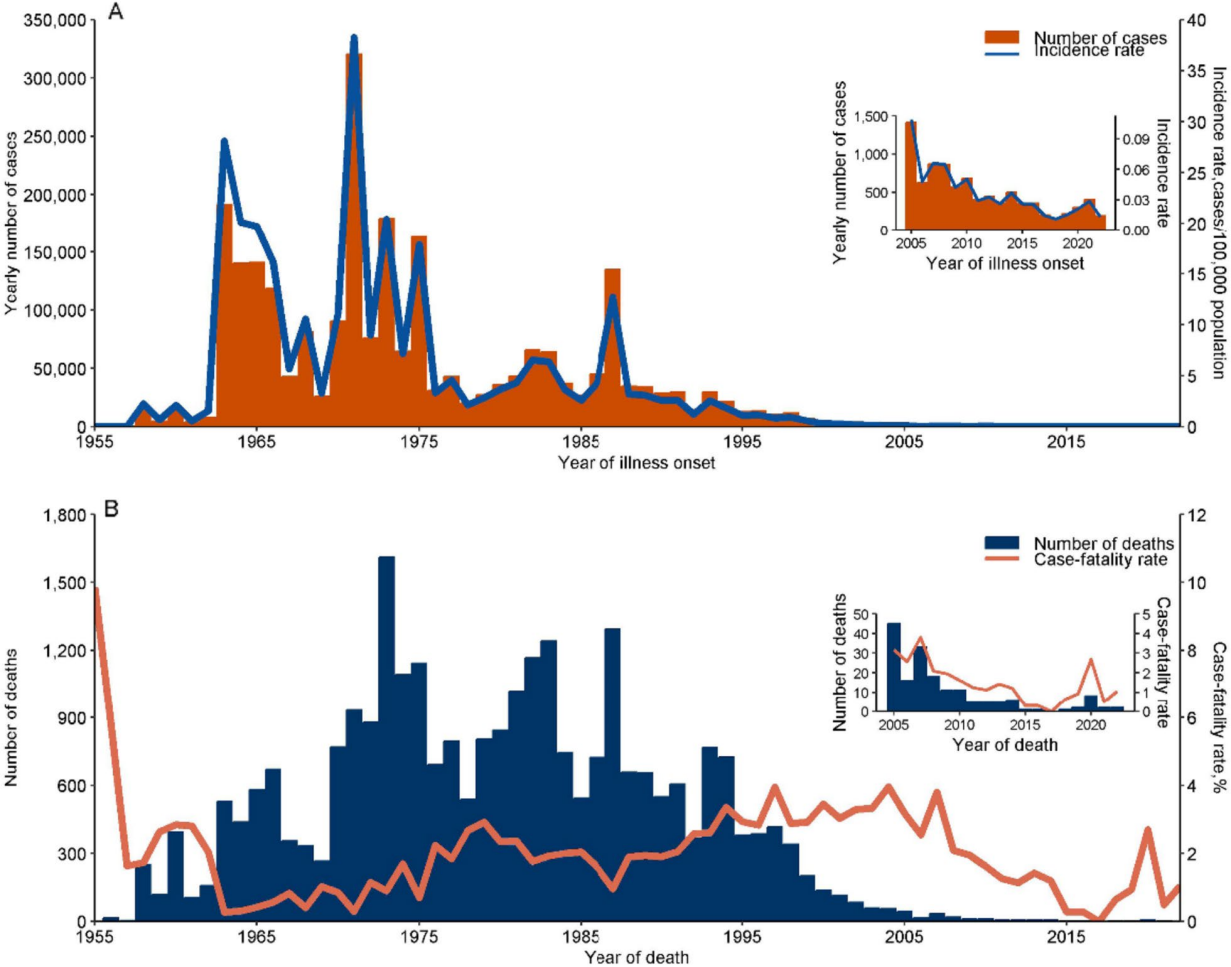
Reported leptospirosis cases and deaths, China, 1955–2022. **A** Number of leptospirosis cases and incidence rate (no. cases/100,000 population) by year. **B** Number of leptospirosis deaths and case-fatality rate (%) by year

To:

**Fig. 1:**
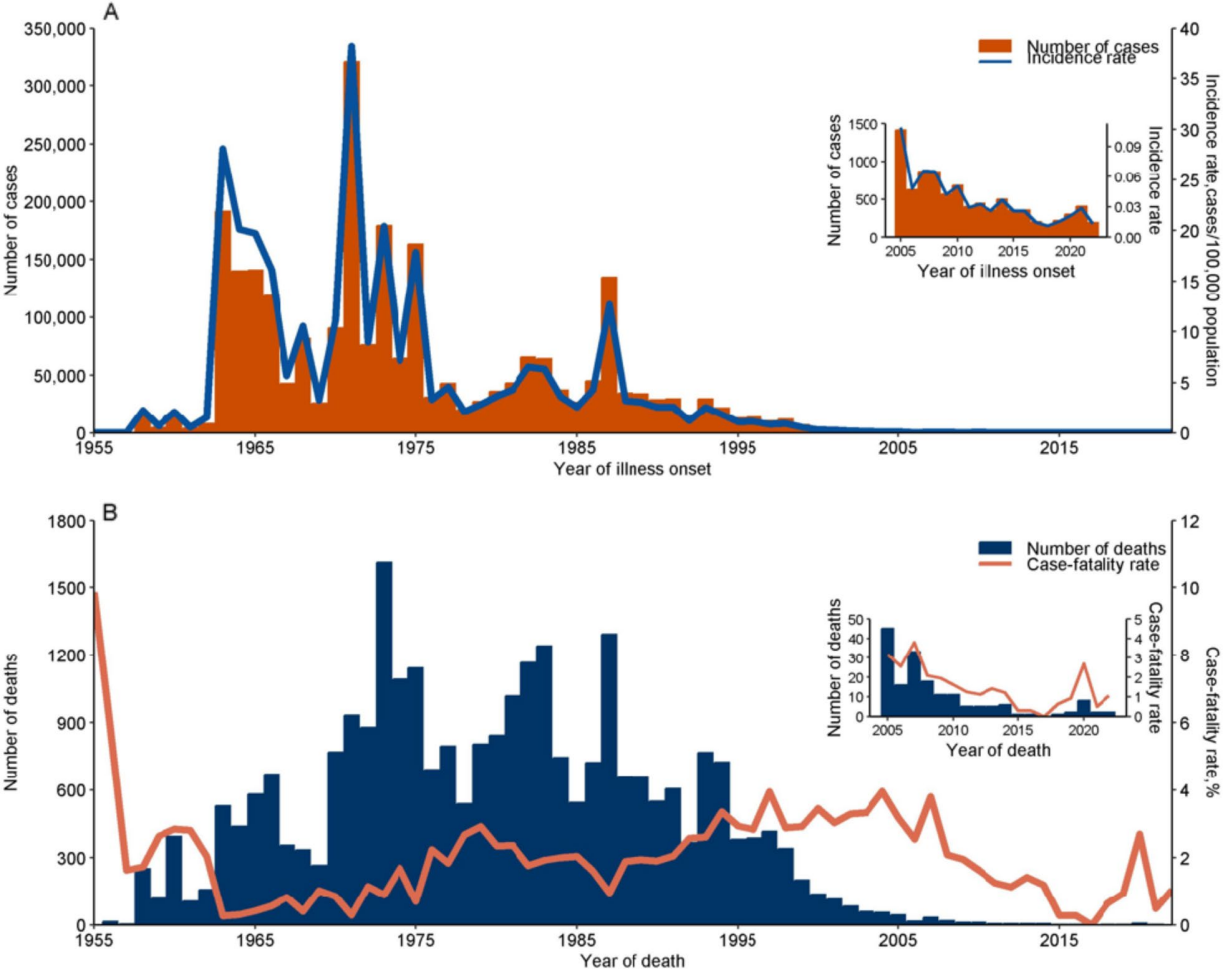
Reported leptospirosis cases and deaths, China, 1955–2022. **A** Number of leptospirosis cases and incidence rate (no. cases/100,000 population) by year. **B** Number of leptospirosis deaths and case-fatality rate (%) by year

In Table 2, 23 cases without address information are included in the Total but excluded from the Northern and Southern categories. To clarify this distinction, we annotated the Total with an asterisk (*).

Table 2 was updated from:

**Table 2:** Demographic characteristics of patients with human leptospirosis, categorised by northern and southern China, 2005–2022

Characteristic	Northern	Southern	Total
	(*n* = 76)	(*n* = 8868)	(*n* = 8967)
Gender			
Male	46 (60.53)	6206 (69.98)	6268 (69.90)
Female	30 (39.47)	2662 (30.02)	2699 (30.10)
Age, years			
Median (Q1, Q3)	48 (32.00, 58.25)	45 (31.00, 57.00)	45 (31.00, 57.00)
Age group			
0–4	2 (2.63)	18 (0.20)	20 (0.22)
5–14	1 (1.32)	550 (6.20)	551 (6.14)
15–24	6 (7.89)	950 (10.71)	960 (10.71)
25–34	13 (17.11)	1182 (13.33)	1196 (13.34)
35–44	11 (14.47)	1718 (19.37)	1731 (19.30)
45–54	15 (19.74)	1784 (20.12)	1807 (20.15)
55–64	17 (22.37)	1618 (18.25)	1640 (18.29)
≥ 65	11 (14.47)	1048 (11.82)	1062 (11.84)
Occupation			
Farmer	44 (57.89)	6582 (74.22)	6644 (74.09)
Student	1 (1.32)	757 (8.54)	760 (8.48)
Worker	3 (3.95)	266 (3.00)	269 (3.00)
Others	25 (32.89)	1121 (12.64)	1149 (12.81)
Unknown	3 (3.95)	142 (1.60)	145 (1.62)

To:

**Table 2:** Demographic characteristics of patients with human leptospirosis, categorised by northern and southern China, 2005–2022

Characteristic	Northern	Southern	Total*
	(*n* = 76)	(*n* = 8868)	(*n* = 8967)
Gender			
Male	46 (60.53)	6206 (69.98)	6268 (69.90)
Female	30 (39.47)	2662 (30.02)	2699 (30.10)
Age, years			
Median (Q1, Q3)	48 (32.00, 58.25)	45 (31.00, 57.00)	45 (31.00, 57.00)
Age group			
0–4	2 (2.63)	18 (0.20)	20 (0.22)
5–14	1 (1.32)	550 (6.20)	551 (6.14)
15–24	6 (7.89)	950 (10.71)	960 (10.71)
25–34	13 (17.11)	1182 (13.33)	1196 (13.34)
35–44	11 (14.47)	1718 (19.37)	1731 (19.30)
45–54	15 (19.74)	1784 (20.12)	1807 (20.15)
55–64	17 (22.37)	1618 (18.25)	1640 (18.29)
≥ 65	11 (14.47)	1048 (11.82)	1062 (11.84)
Occupation			
Farmer	44 (57.89)	6582 (74.22)	6644 (74.09)
Student	1 (1.32)	757 (8.54)	760 (8.48)
Worker	3 (3.95)	266 (3.00)	269 (3.00)
Others	25 (32.89)	1121 (12.64)	1149 (12.81)
Unknown	3 (3.95)	142 (1.60)	145 (1.62)

*The Total includes 23 cases without address information

These corrections do not alter the study’s primary data, analytical methods, or conclusions.

The original article [1] has been updated.
