# Supplementary material for: Changing epidemiology of leptospirosis in China from 1955 to 2022
Source: Infect Dis Poverty. 2025 Mar 3;14:17. doi: 10.1186/s40249-025-01284-x (PMC11874624; doi:10.1186/s40249-025-01284-x)
Supplement: Supplementary file 1 — Supplementary Material 1. [file 40249_2025_1284_MOESM1_ESM.docx]

**Changing Epidemiology of Leptospirosis in China,1955-2022**

**Additional file: Table S1. Variables in the aggregated dataset of leptospirosis cases, China, 1955-2022**

| **Variables** | **Aggregated** | **Period** |
| --- | --- | --- |
| Total cases and no. fatalities | By month at national level | 1955-2022 |
|  | By month at provincial level | 1980-2022 |
|  | By province | 1955-2022 |
|  | By patient gender | 2005-2022 |
|  | By age group | 2005-2022 |
|  | By occupation | 2005-2022 |
| Incidence rate and death rate (per 100,000 residents) | At national level | 1955-2022 |
|  | By province | 1955-2022 |
| The case-fatality rate | At national level | 1955-2022 |
|  | By province | 1955-2022 |

**Additional file: Table S2.Space-time scanning analysis of reported cases of Leptospirosis in China, 1955-2022**

| **Time** | **Cluster** | **Location** | **Radius(km)** | **Period** | **LLR** | ***P*** |
| --- | --- | --- | --- | --- | --- | --- |
| 1955-1964 | 1 | Hebei | 0 | 1963-1963 | 489783.85 | <0.001 |
|  | 2 | Liaoning | 0 | 1964-1964 | 62106.70 | <0.001 |
|  | 3 | Shaanxi | 0 | 1963-1964 | 59183.22 | <0.001 |
|  | 4 | Shandong | 0 | 1964-1964 | 28870.73 | <0.001 |
|  | 5 | Henan | 0 | 1964-1964 | 25527.55 | <0.001 |
|  | 6 | Zhejiang | 0 | 1960-1960 | 471.60 | <0.001 |
|  | 7 | Guizhou | 0 | 1964-1964 | 23.53 | <0.001 |
|  | 8 | Fujian | 0 | 1964-1964 | 16.19 | <0.001 |
| 1965-1974 | 1 | Henan | 0 | 1971-1971 | 302648.95 | <0.001 |
|  | 2 | Anhui | 0 | 1971-1973 | 269386.29 | <0.001 |
|  | 3 | Zhejiang | 0 | 1965-1966 | 61309.03 | <0.001 |
|  | 4 | Shandong | 0 | 1970-1970 | 41209.32 | <0.001 |
|  | 5 | Hubei | 0 | 1973-1973 | 35892.64 | <0.001 |
|  | 6 | Hunan,Jiangxi | 240.48 | 1973-1973 | 17391.74 | <0.001 |
|  | 7 | Guangdong | 0 | 1968-1968 | 8035.31 | <0.001 |
|  | 8 | Liaoning | 0 | 1967-1969 | 5769.54 | <0.001 |
|  | 9 | Shaanxi | 0 | 1965-1965 | 2891.90 | <0.001 |
|  | 10 | Guizhou | 0 | 1973-1974 | 787.40 | <0.001 |
| 1975-1984 | 1 | Henan | 0 | 1975-1975 | 220929.46 | <0.001 |
|  | 2 | Guizhou,Sichuan | 423.07 | 1980-1984 | 102456.28 | <0.001 |
|  | 3 | Hubei | 0 | 1982-1983 | 82759.19 | <0.001 |
|  | 4 | Shandong | 0 | 1975-1975 | 3351.77 | <0.001 |
|  | 5 | Guangdong | 0 | 1975-1976 | 1817.64 | <0.001 |
|  | 6 | Zhejiang | 0 | 1980-1980 | 1373.75 | <0.001 |
|  | 7 | Fujian,Jiangxi | 477.77 | 1975-1975 | 1208.10 | <0.001 |
|  | 8 | Anhui | 0 | 1975-1975 | 646.15 | <0.001 |
|  | 9 | Hebei | 0 | 1977-1977 | 195.00 | <0.001 |
| 1985-1994 | 1 | Sichuan | 0 | 1987-1987 | 256273.43 | <0.001 |
|  | 2 | Hunan,Jiangxi | 240.48 | 1987-1991 | 27537.88 | <0.001 |
|  | 3 | Hubei | 0 | 1986-1986 | 10988.59 | <0.001 |
|  | 4 | Guizhou | 0 | 1985-1987 | 8645.37 | <0.001 |
|  | 5 | Zhejiang | 0 | 1986-1989 | 3339.38 | <0.001 |
|  | 6 | Guangxi | 0 | 1994-1994 | 146.05 | <0.001 |
| 1995-2004 | 1 | Hunan,Jiangxi | 240.4794 | 1995-1999 | 41442.80 | <0.001 |
|  | 2 | Sichuan,Chongqing | 258.2188 | 1995-1999 | 4700.72 | <0.001 |
|  | 3 | Yunnan,Guizhou | 471.9891 | 1995-1998 | 685.36 | <0.001 |
|  | 4 | Guangxi | 0 | 1995-1998 | 543.70 | <0.001 |
| 2005-2014 | 1 | Sichuan | 0 | 2005-2009 | 1788.63 | <0.001 |
|  | 2 | Yunnan,Guizhou | 584.5149 | 2005-2009 | 520.75 | <0.001 |
|  | 3 | Hunan,Jiangxi | 240.4794 | 2005-2008 | 244.96 | <0.001 |
|  | 4 | Anhui | 0 | 2007-2008 | 13.12 | <0.001 |
|  | 5 | Zhejiang | 0 | 2007-2007 | 12.09 | <0.001 |
|  | 6 | Guangdong | 0 | 2005-2008 | 11.81 | <0.001 |
| 2015-2022 | 1 | Yunnan | 0 | 2015-2016 | 388.86 | <0.001 |
|  | 2 | Zhejiang | 0 | 2019-2022 | 201.24 | <0.001 |
|  | 3 | Fujian | 0 | 2015-2018 | 99.15 | <0.001 |
|  | 4 | Hunan | 0 | 2019-2021 | 86.96 | <0.001 |
|  | 5 | Sichuan | 0 | 2019-2021 | 64.33 | <0.001 |
|  | 6 | Anhui | 0 | 2016-2016 | 30.32 | <0.001 |
|  | 7 | Guangdong | 0 | 2021-2022 | 22.37 | <0.001 |
|  | 8 | Guizhou | 0 | 2015-2015 | 8.75 | 0.02 |

**Additional file: Table S3. Descriptive Statistics and Correlation Matrix.**

| **Variable** | ***M*** | ***SD*** | **1** | **2** | **3** | **4** | **5** | **6** |
| --- | --- | --- | --- | --- | --- | --- | --- | --- |
| 1. GDP per capita | 20242.63 | 30364.63 | — |  |  |  |  |  |
| 2. Total power of agricultural machinery | 1553.86 | 2158.00 | 0.36^***^ | — |  |  |  |  |
| 3. Annual average temperature | 11.17 | 6.44 | 0.14^***^ | 0.09^***^ | — |  |  |  |
| 4. Relative humidity | 66.78 | 11.16 | –0.01 | 0.01 | 0.82^***^ | — |  |  |
| 5. Daily sunshine hours | 6.11 | 1.41 | –0.13^***^ | –0.12^***^ | –0.71^***^ | –0.86^***^ | — |  |
| 6. Annual average precipitation | 1109.04 | 563.09 | –0.06^**^ | –0.11^***^ | 0.71^***^ | 0.86^***^ | –0.86^***^ | — |
| 7. Elevation | 927.87 | 1117.75 | –0.13^***^ | –0.16^***^ | –0.69^***^ | –0.61^***^ | 0.35^***^ | –0.30^***^ |

*Note*. *M*:mean, *SD*:standard deviation;* *p* < .05. ** *p* < .01. *** *p* < .001.
