# Supplementary figures and images for: Changing epidemiology of leptospirosis in China from 1955 to 2022
Source: Infect Dis Poverty. 2025 Mar 3;14:17. doi: 10.1186/s40249-025-01284-x (PMC11874624; doi:10.1186/s40249-025-01284-x)

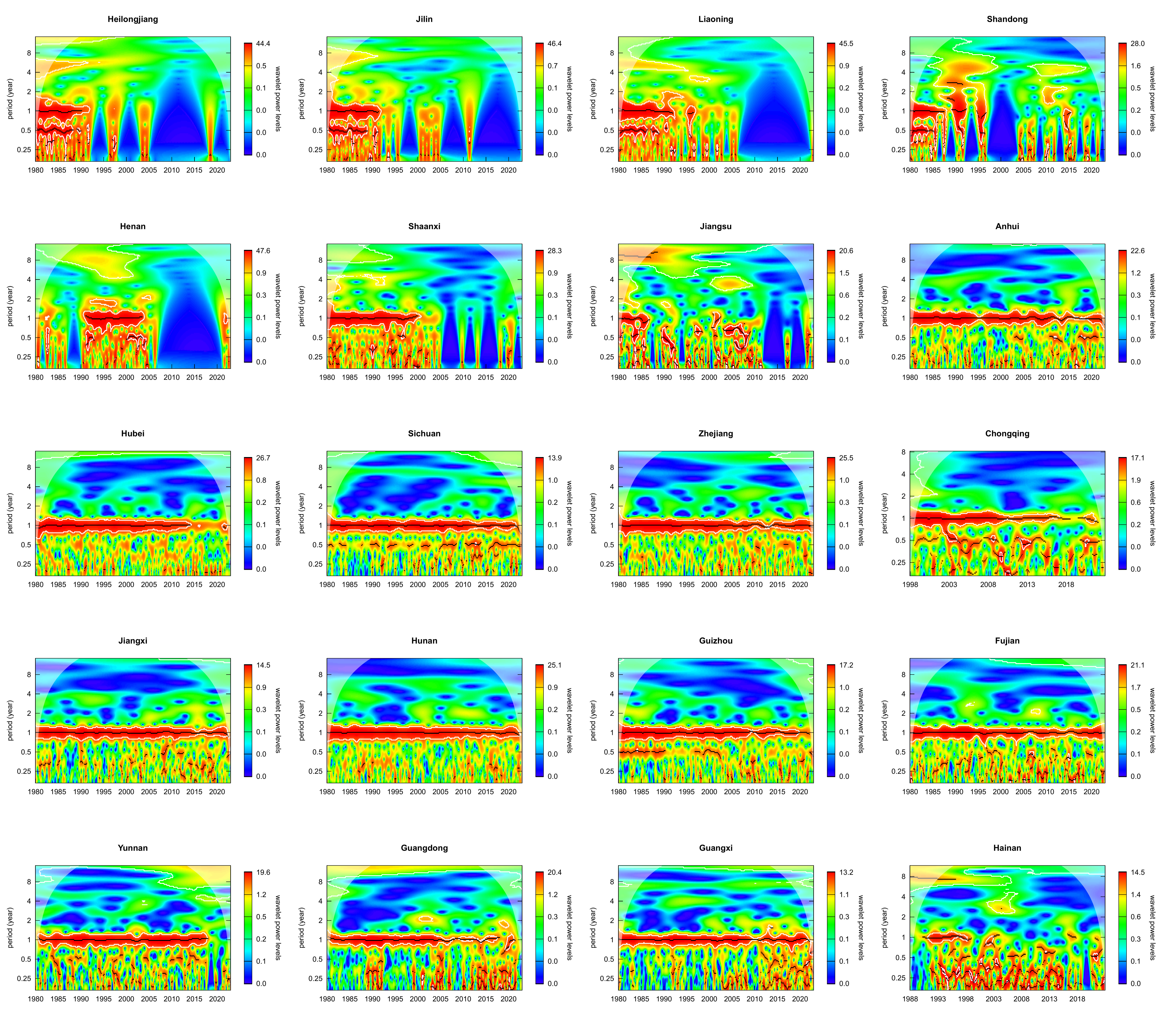

Supplement: Supplementary file 2 — Supplementary Material 2. [file 40249_2025_1284_MOESM2_ESM.jpeg]

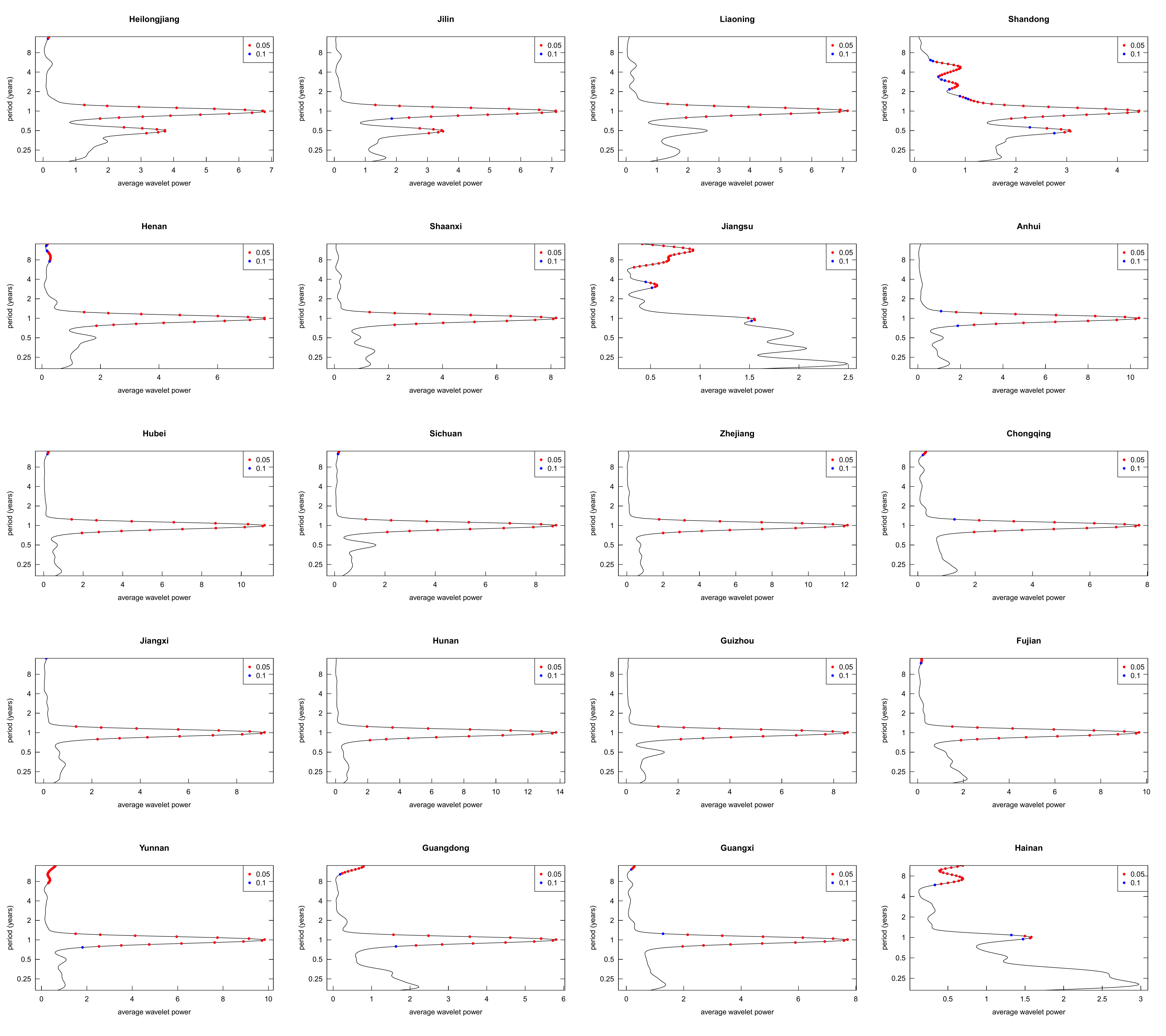

Supplement: Supplementary file 3 — Supplementary Material 3. [file 40249_2025_1284_MOESM3_ESM.jpeg]
